# Identification of the Remains of an Adult Using DNA from Their Deciduous Teeth as a Reference Sample

**DOI:** 10.3390/medicina59101702

**Published:** 2023-09-23

**Authors:** María-de-Lourdes Chávez-Briones, Gilberto Jaramillo-Rangel, Adriana Ancer-Arellano, Jesús Ancer-Rodríguez, Marta Ortega-Martínez

**Affiliations:** Department of Pathology, School of Medicine, Autonomous University of Nuevo Leon, Monterrey 64460, Mexico; mdlourdes.chavezbrn@uanl.edu.mx (M.-d.-L.C.-B.); gilberto.jaramillorn@uanl.edu.mx (G.J.-R.); adar7035@gmail.com (A.A.-A.); ancerrodriguezj@gmail.com (J.A.-R.)

**Keywords:** dental pulp, DNA analysis, human remains, reference sample, teeth, victim identification

## Abstract

In many forensic cases, the identification of human remains is performed by comparing their genetic profile with profiles from reference samples of relatives, usually the parents. Here, we report, for the first time, the identification of the remains of an adult using DNA from the person’s deciduous teeth as a reference sample. Fragments of a skeletonized and burned body were found, and a short tandem repeat (STR) profile was obtained. A woman looking for her missing son went to the authorities. When the DNA profile of the woman was compared to a database, a positive match suggested a first-degree kinship with the person to whom the remains belonged. The woman had kept three deciduous molars from her son for more than thirty years. DNA typing of dental pulp was performed. The genetic profiles obtained from the molars and those from the remains coincided in all alleles. The random match probability was 1 in 2.70 × 10^21^. Thus, the remains were fully identified. In the routine identification of human remains, ambiguous STR results may occur due to the presence of null alleles or other mutational events. In addition, erroneous results can be produced by false matches with close family members or even with people who are completely unrelated to the victim, such that, in some cases, a probability of paternity greater than 99.99% does not necessarily indicate biological paternity. Whenever possible, it is preferable to use reference samples from the putative victim as a source of DNA for identification.

## 1. Introduction

One of the main tasks of legal systems in the investigation of criminal cases is the personal identification of unknown human remains. To achieve this purpose, collaboration between forensic anthropologists, pathologists, and odontologists may be crucial. Also, DNA profiling can be used in the identification of skeletonized or highly decomposed human remains. Identification is usually carried out by comparing the genetic profile from the remains with the genotypes of reference samples from relatives, most commonly the parents of the victim. However, in these cases, ambiguous results may occur due to the presence of null alleles or other mutational events, and erroneous results can be produced by false matches with close family members or even with people who are completely unrelated to the victim [1,2].

Thus, in the identification of human remains by DNA typing, it would be ideal to use biological samples of the person from whom the remains are suspected to have come as a reference. However, there are few reports in the literature on the successful use of this strategy. Calacal et al. [3] identified the skeletal remains of two children by directly comparing the genetic profiles derived from the remains with the profiles from children’s umbilical tissues, which had been preserved by their mothers. Tanaka et al. [4] identified two corpses in two criminal cases using the toothbrushes of the victims as DNA sources. Sweet et al. [5] identified a skeleton using a reference sample consisting of cytological smears stained with the Papanicolaou method, obtained from the medical record of the deceased. Other studies have analyzed the feasibility of using objects or samples from the victim, such as cosmetic applicators [6] and archived tumor samples [7], to obtain DNA that can be used to identify human remains; however, these strategies have not been applied in actual criminal cases.

While bloodstains or buccal swabs would be the perfect reference samples from the victim for the identification of unknown remains, they are often not available. Here, we report, for the first time, the use of DNA isolated from deciduous teeth as a reference sample to identify an adult victim in an actual criminal case.

## 2. Case Presentation

Fragments of a skeletonized and burned body were found on the slopes of a hill. Four of the least damaged bone fragments were selected for DNA extraction. Given the physical condition of the body, we could neither determine to which bones the analyzed fragments belonged nor characteristics such as the sex or the approximate age of the deceased. The outer surfaces of the fragments were cleaned by immersion in 50% commercial bleach for 15 min. Next, they were washed briefly with nuclease-free water (5 washes), then immersed briefly in 100% ethanol and air-dried overnight in a sterile hood. The samples were frozen with liquid nitrogen and pulverized with a pestle and mortar. The bone powder (0.5 g) was decalcified by incubating it with a 0.5 M EDTA solution on a rocking platform at 37 °C for 5 days with three solution changes. Samples were centrifuged, and the pellets were rinsed twice in double-distilled water. DNA extraction was performed using the PrepFiler Express BTA™ Forensic DNA Extraction Kit (Applied Biosystems, Foster City, CA, USA). Lysis buffer from the kit was added to the samples together with 1 M DTT and Proteinase K (2 mg/mL). Samples were incubated overnight in a thermal shaker at 56 °C. Finally, they were centrifuged, and the supernatant was subjected to DNA extraction in the AutoMate™ Instrument (Applied Biosystems) following the manufacturer’s instructions. The DNA samples were quantified on the 7500 ABI Real-Time PCR platform using the Quantifiler Trio DNA quantification kit (Applied Biosystems). All samples had DNA concentrations > 0.01 ng/µL and were therefore deemed suitable for DNA typing [8].

DNA typing was carried out using the commercially available multiplex kit AmpFℓSTR^®^ Identifiler Plus (Applied Biosystems), following the protocol provided by the manufacturer. In an attempt to ensure the amplification of as many alleles as possible, the samples were also amplified by the AmpFℓSTR^®^ MiniFiler kit (Applied Biosystems), which has nine loci in common with the previous kit. Capillary electrophoresis was performed in an ABI PRISM^®^ 310 genetic analyzer (Applied Biosystems). Samples were run on a capillary containing POP-4 polymer; allele assignment was determined by comparison with allelic ladders included in the kits, and genotypes were generated using GeneMapper^®^ IDX-v1.4 software (Applied Biosystems).

No alleles from bone fragment number 1 were amplified. Partial consensus profiles (combining the results of both kits) were obtained from fragments 2 and 3. A complete consensus profile was obtained from fragment 4. The genetic profile obtained was stored in a database containing genotypes from unidentified cadavers.

**Figure 1:**
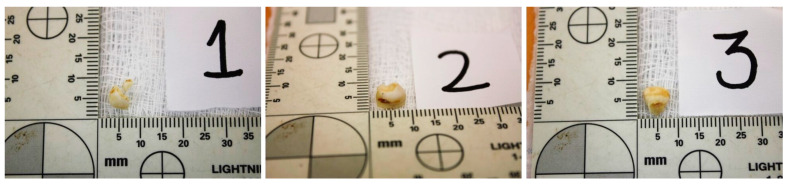
Deciduous molars submitted for DNA typing.

Four years later, a woman looking for her missing son went to the authorities. A saliva sample was obtained according to the usual protocol followed in these cases at our institution. DNA was extracted from the sample using a Chelex protocol [9]. DNA typing was performed as described above for the bone fragments, but only with the Identifiler kit. When the woman’s DNA profile was compared to the database, a positive match suggested a first-degree kinship with the person to whom the remains belonged. The woman was asked about the existence of other first-degree relatives of her son, which could allow a complete identification of the remains. She denied the availability of the father and other first-degree relatives of her son. Later in the interview, she recalled that she had kept three deciduous molars from her son in a plastic bag for more than thirty years (Figure 1). She was asked for the molars to see whether they could serve as a reference sample.

Dental pulp tissue was collected from each molar by sectioning using a carborundum disc. DNA was isolated by Proteinase K digestion and phenol chloroform extraction methods [10] and quantified as described for the bone fragments. DNA typing was performed with the Identifiler and MiniFiler kits as described above. A complete consensus profile was obtained from molar 1. A partial consensus profile was obtained from molar 2. No alleles from molar 3 were amplified.

All the genetic profiles generated are presented in Table 1. The genetic profiles obtained from the bone fragments and the molars coincided in all the alleles. Every locus was sequenced from the bone fragments, and the molars shared at least one allele with the corresponding locus generated from the putative mother. The random match probability and the probability of parentage were calculated using STR allele frequency data from our population and PATPCR software version 2.0.2 [11,12]. The random match probability was 1 in 2.70 × 10^21^, and the probability of parentage was 99.9999%. Thus, the remains were fully identified and returned to the victim’s biological mother.

## 3. Discussion

Dental pulp is a rich source of DNA amenable to genetic analysis; the latter can be used for the positive identification of human remains, especially when soft tissue destruction has occurred. DNA analysis is usually carried out by comparing the genetic profile of the teeth from the remains with the genotypes of reference samples from relatives, most commonly the parents of the victim. For purposes such as crime solving, missing-person cases, and disaster victim identification, this approach has been used for decades [13]. However, to our knowledge, this is the first report of the use of DNA isolated from teeth as a reference sample to identify a victim in a criminal case.

In the case presented here, unambiguous identification was achieved thanks to the matching of DNA profiles generated from the bone fragments with those from the teeth. The DNA profile from the mother served to reinforce the results.

Short tandem repeats (STRs) are the most widely used genetic markers for human identity determination and paternity testing. Their use makes it possible to clarify most legal and forensic cases with a generally very high degree of certainty [14]. As mentioned above, the identification of human remains is generally performed by comparing the genetic profile of the remains with that of first-degree relatives, usually the parents. However, ambiguous STR results may occur due to the presence of null alleles or other mutational events (for specific cases, see [15,16,17,18,19,20,21,22,23,24,25,26,27,28]; for studies in populations, see [29,30,31,32,33,34,35,36,37]). STRs have mutation rates ranging from 0 to 7 × 10^−3^, with an average of 2 × 10^−3^ [33,34]. The most frequent mechanism causing these mutations is the slippage of the DNA replication complex during DNA synthesis [30]. In the most common mutations, an STR differs only slightly in its size from its presumed predecessor. The gain or loss of tandem repeats could lead to false maternal or paternal exclusions [30,32]. In addition, erroneous results can be produced by false matches with close family members or even with people who are completely unrelated to the victim, such that, in some cases, a probability of paternity greater than 99.99% does not necessarily indicate biological paternity [30,31,38,39,40,41]. Poetsch et al. [31] investigated how many wrong paternity inclusions could be detected when comparing 13-15 STRs between 336 children and 348 unrelated men. They found that at least one and up to three “second father(s)” could be found for 23 children. In general, the false inclusion rate ranges between 19% and 23% [40]. These problems are being reported more frequently and are most common in cases where only one putative parent is available [31,40,42]. The inclusion of additional autosomal STR loci may assist in clarifying some ambiguous cases. Sometimes, however, the addition of more loci introduces additional mismatches. Furthermore, it has been observed that the inclusion of more loci does not compensate for the absence of genetic information from the mother or the father [35,40,42,43,44]. The use of Y-chromosome STRs can help only when the victim is male, and the possibility that a close relative of the putative father is the biological father cannot be ruled out [45]. X-chromosome STRs must be analyzed along with other genetic markers to obtain useful data and can only be used with accuracy when the victim is female, as there are no X-chromosome alleles inherited by descent in a father-son relationship [46]. Other typing systems that may be used to resolve ambiguous cases include the HV1 and HV2 hypervariable regions of mitochondrial DNA, single nucleotide polymorphisms (SNPs), and next-generation sequencing (NGS). However, they are expensive and time-consuming and are not available in most developing countries [47]. Even with these systems, the lack of informative reference samples (first-degree relatives) is the most common problem in identifying unknown corpses [41,48]. Thus, whenever possible, it is preferable to use reference samples from the putative victim as a source of DNA for identification.

In this study, we analyzed three deciduous molars. A complete DNA profile was obtained from only one molar. The efficiency of DNA typing from teeth subjected to various experimental conditions, such as treatment with acids [49] and fire exposure [50,51], has been reported in the literature. In addition, the effect of the duration of the postmortem and postextraction periods in obtaining genetic profiles from the teeth has been analyzed [52,53]. From these and other studies, it can be concluded that the usefulness of teeth to obtain a genetic profile not only depends on the conditions to which they are subjected before analysis but also varies between individuals and even within the same individual. This inter- and intra-individual variation may be due to a wide difference in the number of cells present in each individual tooth, resulting in a different DNA yield. In turn, the different number of cells is due to various factors, including the presence or absence of disease and the age of the subject. Therefore, each identification case must be considered individually [53,54].

It is important to note that the mother’s decision to keep some teeth from her son was essential for the resolution of this case. This and similar practices [55] should be promoted, as teeth can be an alternative source of reference DNA for the identification of persons in, for example, mass disasters or criminal cases. Other samples may also be considered for this purpose, such as buccal swabs, hair, and blood spots. Instructions for their collection and preservation, as well as the material required even in a domestic setting, can be easily found on the Internet. However, in this regard, one must be very careful and sensitive and respect the customs and beliefs of a particular society or individual.

Finally, although DNA profiling is an important element for the identification of human remains, several factors can affect the results of this analysis, such as an insufficient amount of extracted DNA or its degradation in cases of poorly preserved samples. In such cases, a multidisciplinary approach may be necessary that considers the use of other disciplines, including forensic anthropology and odontology [56,57].

## 4. Conclusions

This is the first reported case of the use of DNA isolated from teeth as a reference sample to identify a victim in a criminal case. Whenever possible, it is preferable to use reference samples from the putative victim as a source of DNA for identification.

## Figures and Tables

**Table 1:** Comparison of short tandem repeats results of DNA recovered from bone fragments, deciduous molars, and the alleged mother of the victim.

Locus	Bone Fragments	Deciduous Molars	Alleged Mother
Amelogenin	XY	XY	XX
D8S1179	14, 15	14, 15	12, 14
D21S11	32.2, 33.2	32.2, 33.2	29, 33.2
D7S820	10, 10	10, 10	10, 10
CSF1PO	10, 11	10, 11	11, 11
D3S1358	15, 18	15, 18	14, 15
TH01	6, 6	6, 6	6, 6
D13S317	9, 14	9, 14	9, 9
D16S539	11, 12	11, 12	11, 12
D2S1338	18, 25	18, 25	23, 25
D19S433	11.2, 13	11.2, 13	11.2, 15
vWA	16, 17	16, 17	16, 17
TPOX	9, 12	9, 12	8, 9
D18S51	12, 15	12, 15	15, 16
D5S818	11, 12	11, 12	11, 11
FGA	21, 24	21, 24	24, 24

## Data Availability

All data generated or analyzed for this report are included in the published article.
